# Aberrant activation of neuronal cell cycle caused by dysregulation of ubiquitin ligase Itch results in neurodegeneration

**DOI:** 10.1038/s41419-020-2647-1

**Published:** 2020-06-08

**Authors:** Monika Chauhan, Prashant Kumar Modi, Pushkar Sharma

**Affiliations:** 1 Eukaryotic Gene Expression Laboratory, National Institute of Immunology, New Delhi, India; 2 Present Address: Center for Systems Biology and Molecular Medicine, Yenepoya Research Centre, Yenepoya (Deemed to be University), Mangalore, 575018 India

**Keywords:** Cell death in the nervous system, Neurological disorders

## Abstract

It is critical for the neuronal cell cycle to remain suppressed in terminally differentiated neurons as its activation results in aberrant cell cycle re-entry that causes neuronal apoptosis (CRNA), which has been observed in several neurodegenerative disorders like Alzheimer’s disease (AD). In the present study, we report that E3 ubiquitin ligase Itch is a major regulator of CRNA and elucidated the mechanism via which it is regulated in this process. Neurotoxic amyloid peptide A*β*_42_-treated neurons or neurons from an AD transgenic mouse model (TgAD) exhibited aberrant activation of the JNK pathway which resulted in the hyperphosphorylation of Itch. The phosphorylation of Itch primes it for autoubiquitination, which is necessary for its activation. These post-translational modifications of Itch facilitate its interaction with TAp73 resulting in its degradation. These series of events are critical for Itch-mediated CRNA and its phosphorylation and autoubiquitination site mutants reversed this process and were neuroprotective. These studies unravel a novel pathway via which neurodegeneration in AD and possibly other related disorders may be regulated by aberrant regulation of the neuronal cell cycle.

## Introduction

It is imperative for the cell cycle of terminally differentiated neurons to remain suppressed in order to maintain their viability. Neurotoxic conditions that include trophic factor withdrawal, encounter with misfolded proteins like beta amyloid peptide A*β*_42_ and DNA damaging agents are known to trigger aberrant re-entry into the cycle^1,2^. Instead of resulting in mitosis, it causes neuronal cell death^3,4^. One of the key features of CRNA is aberrant regulation of important cell cycle proteins which promote apoptosis. A series of events is triggered by neurotoxic insults resulting in an alarming increase in Cyclin D1 causing activation of CDK4/6, retinoblastoma phosphorylation and transcriptional activation of E2F^4,5^. As a result, neurons exhibit S-phase entry and DNA replication but most of these neurons undergo apoptosis^3,6^. Aberrant cell cycle re-entry and neuronal loss has been observed in several neurodegenerative disorders like Alzheimer’s disease (AD) in which neuronal loss and aberrant cell cycle re-entry are coincidental^7–11^.

Studies performed in vitro as well as in animal models of AD suggest that A*β*_42_ causes cell cycle re-entry and DNA replication that precedes cell death^10,12,13^. Cyclin D1 plays a critical role in this process as it is an upstream regulator of the cascade^14,15^ and its expression aberrantly increases and causes S-phase entry of neurons^13^. Previously, we demonstrated that hyper activation of the MEK-ERK pathway results in enhanced Cyclin D1 production^13^ and temporal down regulation of microRNA-34a, which targets Cyclin D1, resulted in aberrant increase in its expression^12^. TAp73 regulates expression of miR-34a which is important for neuronal differentiation and neurite outgrowth^16^. We demonstrated that TAp73 undergoes ubiquitination in response to A*β*_42_, which was the cause of its degradation^12^ and triggered aberrant cell cycle re-entry and apoptosis of neurons.

p53-family transcription factors p73 can be synthesized in at least seven isoforms of TAp73, which are mainly generated by alternative splicing at the 3′-end^17^. In addition, an alternate promoter and extra exon are used to generate N-terminal truncated versions of the full-length protein (ΔNp73)^18^. These truncated versions lack the N-terminal transactivation (TA) domain, which can block the function of full-length TAp73^19,20^. The relative expression of these two isoforms regulates cell fate^21,22^. Mice lacking either of these isoforms exhibit brain defects that include hippocampal dysgenesis, neurodegeneration, and genomic instability^23,24^.

We have identified Itch—a Nedd4 family E3 Ubiquitin ligase^25,26^ as a regulator of CRNA, which it achieves by promoting degradation of TAp73 in neurons. Thus far, Itch has mainly been implicated in chronic inflammation, T-cell response and other immunological functions. *Itchy* mice that have an inversion in Itch locus exhibit aberrant scratching and immune functions and inflammation^27^. Itch deficiency causes multi-system autoimmune disease^28^ and Itch-deficient mice exhibit chronic production of tumorigenic cytokines and exhibited higher propensity for tumor formation^26,29,30^. Several Itch targets have been identified in immune cells, which include c-jun, E3 ligase Cbl and TAp63, and TAp73^25,31–33^. Itch facilitates the degradation of p63 and p73 but not p53, which may affect tumor cell response^29,30^. RASSF5, a downstream effector of Ras involved in cell cycle arrest, is also targeted by Itch^34^. Itch is regulated by post-translation modifications like phosphorylation^32,33^, autoubiquitination^35,36^. Stress-induced JNK activation results in the phosphorylation of Itch in an N-terminal proline-rich region (PRR), which induces a conformational change facilitating its autoubiquitination at specific sites^32,33^.

Despite these studies, the role of Itch in neuronal development or neurodegeneration has remained almost unknown. In our quest to dissect mechanisms involved in TAp73 degradation during CRNA, we identified Itch as the candidate E3-ligase. We found that A*β*_42_ activates Itch in neurons by promoting its phosphorylation via the JNK pathway, which further facilitates its autoubiquitination at specific sites. These events prime Itch to interact with TAp73 in TgAD neurons resulting in degradation of the latter. As a result, neurons undergo aberrant cell cycle re-entry and apoptosis. We generated mutants of Itch defective in phosphorylation and autoubiquitination, which reversed the CRNA of TgAD neurons by preventing TAp73 degradation.

## Results

### Itch regulates the degradation of TAp73 mediated by A*β*_42_

TAp73 is critical for neuronal differentiation and survival^16,37^. Previously, we demonstrated that it is ubiquitinated and degraded in neurons upon treatment with neurotoxic A*β*_42_^12^, which promoted cell cycle re-entry and apoptosis. In order to get deeper insights into the underlying mechanisms, it was pertinent to identify the E3 Ub-ligase is involved in TAp73 degradation.

**Fig. 1:**
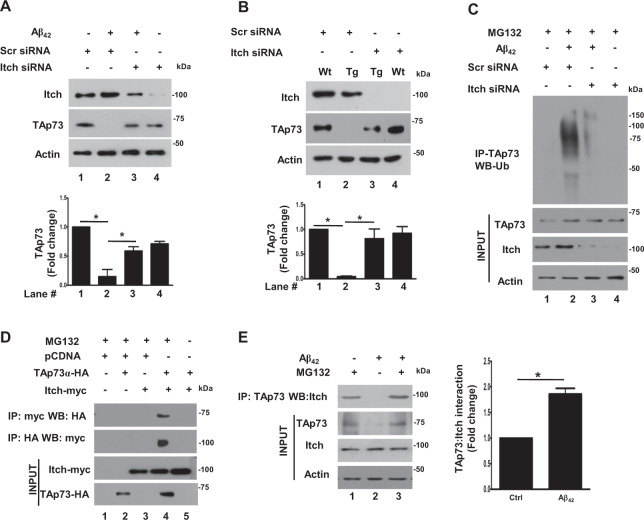
Itch regulates A*β*_42_-mediated degradation of TAp73. **a** Rat cortical neurons, which were transfected with siRNA against Itch or a scrambled siRNA, scr_siRNA (control) and were treated with A*β*_42_ for 48 h or left untreated. Subsequently, Western blotting was performed for assessing the expression of TAp73, Itch, or Actin (loading control). Itch depletion significantly prevented TAp73 degradation, which was quantified by densitometry of TAp73 bands (bottom panel), which was normalized with respect to Actin (mean ± SEM, ANOVA, *p* < 0.05, *N* = 3). **b** Cortical neurons were cultured from wild type (WT) or APP/PS1 transgenic (Tg) mice and transfected with Itch siRNA or scrambled scr_siRNA (control). After 48 h, cell lysate was prepared and Western blotting was performed for TAp73, Itch or Actin. Itch depletion significantly prevented TAp73 degradation that was quantified by densitometry of TAp73 bands, which was normalized with respect to Actin (mean ± SEM, ANOVA, *p* < 0.05, *N* = 3). **c** Rat cortical neurons, which were transfected with siRNA against Itch or a scrambled siRNA (control), were left untreated or treated with A*β*_42_ for 48 h in the presence of MG132. TAp73 was immunoprecipitated and the IP was used for Western blotting with anti-ubiquitin antibody. **d** Neuronal PC12 cells were transfected with expression plasmids for the overexpression of TAp73-HA or Itch-Myc or control vector (pcDNA) in presence or absence of MG132. After 48 h, cell lysates were prepared and used for immunoprecipitation using anti-HA or anti-Myc antibodies. HA-IP and Myc-IP were immunoblotted with anti-myc and anti-HA, respectively. Immunoblotting was also performed on total cell lysates (input). **e** Rat cortical neurons were treated with A*β*_42_ for 48 h in the presence or absence of proteasome inhibitor MG132. Subsequently, TAp73 was immunoprecipitated followed by Western blotting for Itch. Cell lysates were also immunoblotted to detect expression of these proteins. There was a significant increase in Itch co-immunoprecipitated with TAp73 upon A*β*_42_ treatment, which was also quantified by densitometry of bands corresponding to Itch (right panel, mean ± SEM, *p < 0.05, *t*-test, *N* = 3).

Previous studies performed in non-neuronal HEK293 or HeLa cells indicated that HECT-family ligase Itch is a major mediator of p73 degradation^30^. In order to explore if Itch is involved in TAp73 degradation in neurons in response to A*β*_42_, cortical neurons were treated with A*β*_42_ for 48 h as described previously^12^ in the presence or absence of siRNA against Itch. There was no major change in TAp73 under steady-state conditions. While TAp73 was degraded in response to A*β*_42_, Itch depletion almost completely reversed this process and TAp73 expression was retained (Fig. 1a). Similar experiments were also performed on cortical neurons derived from a mouse model for AD (TgAD) that overexpress mutant forms of amyloid precursor protein (APP) and Presnillin1 (PS1) associated with AD^38,39^, which results in enhanced A*β*_42_ production and CRNA has been observed in these animals^12^. The expression of TAp73 was barely detectable in cortical neurons from TgAD animals in contrast to WT. Strikingly, Itch siRNA, but not a control scrambled siRNA, caused a significant increase in TAp73 (Fig. 1b). The status of TAp73 ubiquitination was assessed by performing IP followed by Western blotting for ubiquitin. As reported previously^12^, A*β*_42_ caused TAp73 ubiquitination, which was markedly suppressed upon Itch depletion (Fig. 1c). Collectively, these results indicated that Itch mediates the ubiquitination and degradation of TAp73 in response to A*β*_42_. Next, the association of Itch with TAp73 was tested in neuronal cells, which has not been established as yet. To this end, TAp73 and Itch were overexpressed in neuronal PC12 cells followed by immunoprecipitation (IP). TAp73, co-immunoprecipitated with Itch and vice versa indicating that they interact with each other (Fig. 1d). The effect of A*β*_42_ on their association was tested in cortical neurons by performing co-IP experiments. While endogenous Itch and TAp73 interacted in untreated neurons, there was a significant increase upon A*β*_42_ treatment (Fig. 1e). These results explained enhanced degradation of TAp73 in an Itch-dependent manner (Fig. 1a) and established that A*β*_42_ promotes association of Itch with TAp73 leading to its ubiquitination and degradation.

### Itch promotes cell cycle re-entry and apoptosis of neurons

**Fig. 2:**
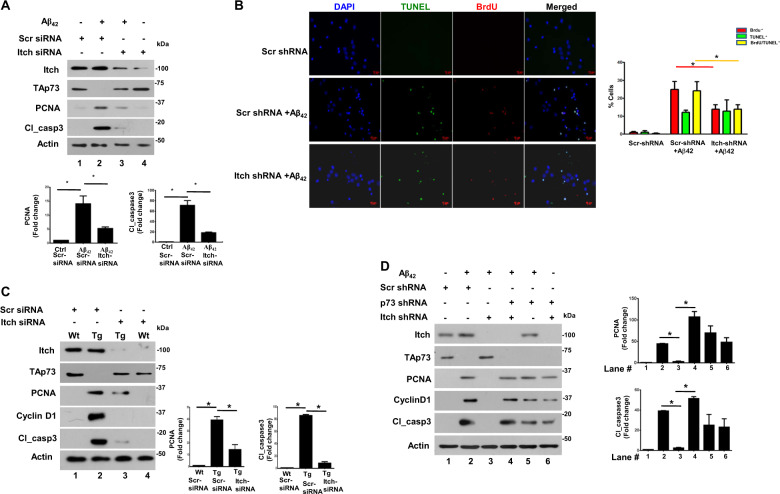
Itch regulates cell cycle-related neuronal apoptosis (CRNA). **a** Rat cortical neurons were transfected with siRNA against Itch or a scrambled control followed by treatment with A*β*_42_ for 48 h. Subsequently, Western blotting was performed for assessing the expression of indicated proteins. Itch depletion significantly prevented TAp73 degradation and blocked the increase in PCNA and cl_caspase3. Bottom panel: Densitometry of PCNA and cl_caspase 3 bands was performed, and their level was normalized with respect to Actin. Fold change in these proteins with respect to untreated neurons transfected with control siRNA was determined (mean ± SEM, *p < 0.05 by ANOVA, *N* = 3). **b** Rat cortical neurons were transfected with Itch shRNA or control scr_shRNA and treated with A*β*_42_ followed by incubation with BrdU. Immunofluorescence and TUNEL assay were performed to detect BrdU incorporation (red) or apoptosis (green), respectively. Right panel, % cells that were BrdU^+^ (red), TUNEL^+^ (green), or were BrdU^+^/TUNEL^+^ (yellow) were determined (mean ± SEM, *p < 0.05, *t*-test, *N* = 3). **c** Cortical neurons were cultured from wild type (WT) or APP/PS1 Transgenic (Tg) mice and transfected with Itch siRNA or scrambled scr_siRNA (control). After 48 h, cell lysate was prepared and Western blotting was performed for indicated proteins. Right panel, Densitometry of PCNA and cl_caspase 3 bands was performed, and their expression was normalized with respect to actin. Fold change in these proteins with respect to WT neurons transfected with control siRNA was determined (mean ± SEM, *p < 0.01 by ANOVA, *N* = 3). **d** Rat cortical neurons were transfected with shRNA against Itch or a scrambled control. In addition, in some cases shRNA against TAp73 was also transduced using lentivirus. Following treatment with A*β*_42_ for 48 h, Western blotting was performed for assessing the expression of indicated proteins. Itch depletion significantly prevented TAp73 degradation and blocked increase in PCNA and cl_caspase3. TAp73 knockdown in Itch-depleted cells restored cl_caspase3 and PCNA expression. Right panel, Densitometry of PCNA and cl_caspase3 bands was performed, and their expression was normalized with respect to Actin. Fold change in these proteins with respect to scr-shRNA-transfected cells was determined (mean ± SEM, *p < 0.05, *t*-test, *N* = 3).

Next, we evaluated the role of Itch in A*β*_42_-induced cell cycle re-entry and apoptosis of neurons. We used two previously reported approaches for this purpose: levels of PCNA that suggest S-phase entry and cleaved caspase 3 (cl_caspase3), which represents the active form of caspase 3 and is indicative of apoptosis, were determined by Western blotting; BrdU incorporation and TUNEL labeling was performed to detect DNA replication and apoptosis at the single cell level, respectively^12,13^. PCNA is present at very low or almost undetectable levels in cortical neurons from E18 embryos suggesting that these cells are terminally differentiated and have exited the cell cycle (Fig. 2a). While A*β*_42_ treatment resulted in a significant increase in both these proteins, which was indicative of CRNA as reported previously^12^. The knockdown of Itch significantly reduced both PCNA and cl_casapse3 suggesting that it regulates CRNA (Fig. 2a). A significant increase in BrdU^+^/TUNEL^+^ neurons was observed upon A*β*_42_ treatment and most BrdU^+^ neurons were also TUNEL^+^, which indicated that those neurons that re-entered the cell cycle also underwent apoptosis and there was a population, which was only TUNEL^+^ but did not exhibit BrdU incorporation, that represented “conventional” apoptosis independent of cell cycle re-entry as described previously^12,13^. However, the knockdown of Itch caused a significant decrease in BrdU^+^/TUNEL^+^ cells whereas TUNEL^+^ cells were almost unaltered (Fig. 2b) suggesting that mainly those neurons that underwent apoptosis as a result of cell cycle re-entry were rescued by Itch depletion. These data suggested that A*β*_42_-mediated Itch regulation results in aberrant cell cycle re-entry and neuronal apoptosis. Next, studies were also carried out in neurons from TgAD animals. The knockdown of Itch in neurons from TgAD caused a significant decrease in PCNA and cleaved caspase3, which were elevated in these cells in comparison to WT neurons (Fig. 2c).

As reported above (Fig. 1a), Itch is involved in TAp73 degradation in response to A*β*_42_. Therefore, in order to investigate if Itch-mediated degradation of TAp73 caused this process; TAp73 was knocked down in combination with Itch depletion. Itch shRNA prevented the TAp73 degradation and CRNA as evidenced by suppressed PCNA and cleaved caspase3 levels (Fig. 2d lane 3 vs. lane 2). Simultaneous addition of TAp73 shRNA reversed this process as levels of both these proteins increased (lane 4 vs. lane 3). Based on these results, it is reasonable to state that Itch can cause CRNA by promoting degradation of TAp73.

### Phosphorylation of Itch by the JNK pathway is critical for its autoubiquitination and interaction with TAp73

Next, we sought the mechanisms via which Itch is regulated in AD models. We did not find a major increase in the expression of Itch mRNA upon A*β*_42_ treatment of cortical neurons or in TgAD mouse neurons (Supplementary Fig. S1). There was only a modest increase in Itch protein levels (Figs. 1a and 2a, c and d). Therefore, we speculated the role of post-translational modifications in regulation of Itch as previous reports had indicated that it can be regulated by post-translational events like autoubiquitination and phosphorylation^32,33,40^.

**Fig. 3:**
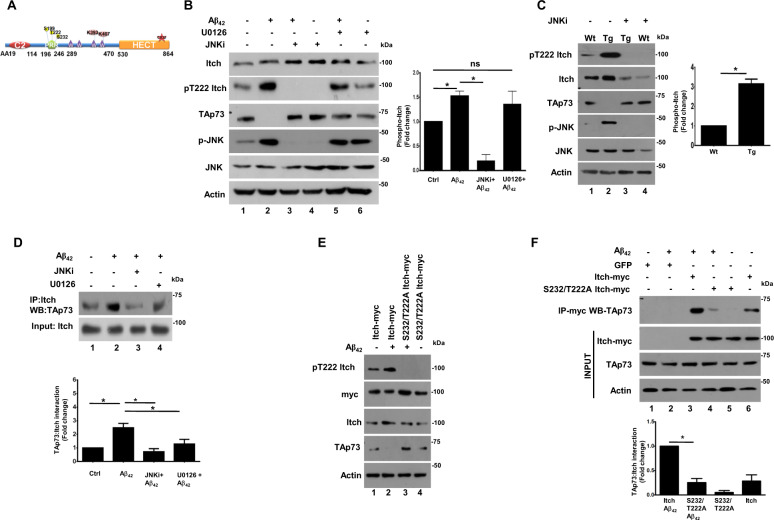
A*β*_42_-induced phosphorylation of Itch via JNK is critical for its interaction with TAp73. **a** Schematic illustrating domain architecture of Itch. In addition to HECT and WW domains, Itch has a proline-rich region (PRR), which is susceptible to phosphorylation at S199, T222, S232. The lysines K383 and K407 implicated in auto-ubiquitination and catalytic C832 are also indicated. **b** Rat cortical neurons were treated with A*β*_42_ for 48 h in the presence or absence of JNK inhibitor (JNKi) or MEK inhibitor (U0126). Cell lysates were prepared and immunoblotted with antibodies specific to Itch phosphorylated at T222, TAp73, and against total JNK and Itch. The level of Itch phosphorylation was quantitated by performing densitometry of phospho-Itch band, which was normalized with respect to total Itch and fold change in phospho-Itch was determined (right panel, mean ± SEM, *p < 0.05, *t*-test, *N* = 4). **c** WT and TgAD mouse cortical neurons were treated with JNK inhibitor (JNKi) for 48 h. Western blotting was performed for phospho-Itch, phospho-JNK and TAp73 as described in panel **b**. Actin was used as a loading control. The level of Itch phosphorylation was quantitated by performing densitometry of phospho-Itch band as described in panel **b** (right panel, mean ± SEM, *p < 0.05, *t*-test, *N* = 3). **d** Rat cortical neurons were treated with A*β*_42_ for 48 h in the presence or absence of JNKi or U0126 in the presence of MG132. Subsequently, Itch was immunoprecipitated followed by Western blotting for TAp73. Cell lysates were also immunoblotted to determine levels of Itch. The increase in Itch co-immunoprecipitated with TAp73 upon A*β*_42_ treatment was significantly reduced upon JNKi or U0126 treatment, which was quantified by densitometry of bands corresponding to TAp73 in Itch-IP (bottom panel, mean ± SEM, *p < 0.05, *t*-test, *N* = 3). **e** Rat cortical neurons were infected with adenovirus to overexpress Itch or its S232/T222A mutant or GFP (control). Subsequently, neurons were treated with A*β*_42_ for 48 h and Western blotting was performed by using antibodies against indicated proteins. S232/T222A mutant was not phosphorylated and it prevented the degradation of TAp73 in A*β*_42_-treated cells. **f** Rat cortical neurons were infected with adenovirus to express Itch or its S232/T222A mutant or GFP (control) in the presence of MG132. Subsequently, A*β*_42_ treatment was given for 48 h followed by immunoprecipitation using anti-myc antibody to IP Itch followed by Western blotting for TAp73. The interaction of S232/T222A mutant was significantly reduced to TAp73, which was also quantified by densitometry of bands corresponding to TAp73 in Itch-IP (bottom panel, mean ± SEM, *p < 0.05, *t*-test, *N* = 3).

Previous reports also indicated that a PRR of Itch (Fig. 3a) is susceptible to phosphorylation by kinases like JNK at S199, T222, S232 which in turn is critical for its activation^32,33^. These sites are proline directed (SP/TP) which are typically targeted by MAP kinases, we speculated a role of MAP kinases like JNK and previous studies have shown that in immune cells JNK can phosphorylate these sites^32^. Therefore, first the role of JNK and ERK in phosphorylation of T222 was evaluated using a specific phospho antibody, which was commercially available. While Itch was phosphorylated at this site in neurons in steady-state conditions, A*β*_42_ caused a significant increase in phosphorylation of T222 (Fig. 3b, lane 1 vs. lane 2). The addition of JNK inhibitor SP600125 (JNKi) caused a dramatic decrease in the phosphorylation of Itch at T222 (lane 3) whereas MEK-ERK inhibitor U0126 did not cause much change (lane 5). Importantly, JNKi also prevented TAp73 degradation by A*β*_42_ suggesting Itch phosphorylation at this site by JNK in response to A*β*_42_. The phosphorylation of Itch at T222 was also higher in neurons derived from TgAD mice; JNKi suppressed the phosphorylation and also prevented TAp73 degradation (Fig. 3c). Further, the role of JNK-induced phosphorylation of Itch and TAp73 interaction was assessed by co-immunoprecipitation (co-IP), which revealed that A*β*_42_-induced interaction of Itch and TAp73 was significantly attenuated by JNK inhibition in both A*β*_42_-treated cortical neurons (Fig. 3d) as well as neurons from TgAD animals (not shown here). JNKi also prevented interaction between JNK and Itch (Supplementary Fig. S4B). These data indicated that JNK pathway triggers phosphorylation of Itch which promotes interaction with TAp73. As mentioned above, we have previously demonstrated that aberrant activation of the MEK-ERK pathway in TgAD or A*β*_42_-treated neurons results in TAp73 ubiquitination-degradation^12^, which was also observed in present studies (Fig. 3b, lane 5 vs. lane 2). Therefore, we tested if this pathway influences TAp73–Itch interaction and co-IP in the presence of U0126 revealed a significant decrease in interaction of these proteins in A*β*_42_-treated neurons (Fig. 3d, lane 4 vs. lane 2). Since MEK-ERK pathway does not seem to contribute to Itch phosphorylation at T222 (Fig. 3b), which is critical for its activation (see below), it is likely that this pathway influences phosphorylation of TAp73 to promote its interaction with Itch, although it needs experimental demonstration.

To further investigate the role of Itch phosphorylation sites, T222 and a neighboring proline-directed site S232, which is also a putative JNK target was mutated to A and this phosphodeficient mutant (T222A/S232A) was overexpressed in cortical neurons using adenovirus. Expectedly, the phospho-T222-Itch antibody did not recognize the T222A/S232A mutant (Fig. 3e). Interestingly, the TAp73 degradation, which was observed in A*β*_42_-treated WT Itch overexpressing neurons (Fig. 3e, lane 2) was almost completely reversed in Itch phospho-deficient mutant overexpressing cells (lane 3). The role of S232/T222 phosphorylation in Itch–TAp73 interaction was also investigated. The interaction of Itch with TAp73 in the presence of A*β*_42_ was significantly impaired upon mutation of T222A/S232A (Fig. 3f). Similar results were obtained in TgAD neurons as both Itch phosphorylation and TAp73 degradation were attenuated upon overexpression of this phospho-deficient mutant (Supplementary Fig. S3). Collectively, these data established that phosphorylation of Itch at T222 and S232 is critical for TAp73 degradation.

### The phosphorylation of Itch promotes its autoubiquitination

**Fig. 4:**
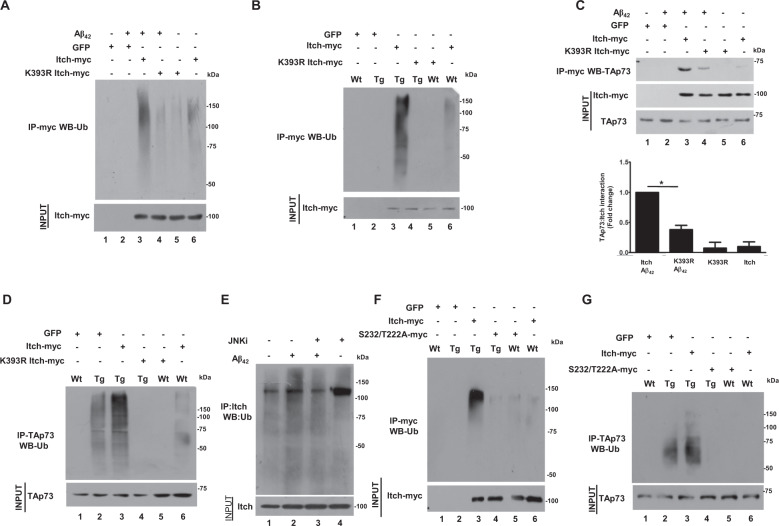
A*β*_42_ promotes autoubiquitination of Itch, which is dependent on its phosphorylation and is critical for interaction with TAp73. **a** Rat cortical neurons were infected with adenovirus to express Itch or its K393R mutant or GFP (control) in the presence of MG132. Subsequently, neurons were treated with A*β*_42_ for 48 h followed by immunoprecipitation using anti-myc antibody to IP Itch, which was immunoblotted for ubiquitin. A significant decrease in ubiquitination of the K393R mutant was observed. **b** Neurons from WT or TgAD mice were infected with adenovirus to express Itch or K393R mutant or GFP (control) in the presence of MG132. Subsequently, immunoprecipitation was performed using anti-myc antibody to IP Itch, which was immunoblotted for ubiquitin. A significant decrease in ubiquitination of the K393R mutant was observed in TgAD neurons. **c** Rat cortical neurons were infected with adenovirus to express Itch or K393R mutant or GFP (control) in the presence of MG132. Subsequently, neurons were treated with A*β*_42_ for 48 h followed by immunoprecipitation using anti-myc antibody to IP Itch followed by Western blotting for TAp73. Densitometry of bands corresponding to TAp73 in Itch-IP was performed for quantitation of interaction and fold change was determined with respect to wild type Itch transduced neurons (bottom panel, mean ± SEM, *p < 0.01, *t*-test, *N* = 3). **d** Neurons from WT or TgAD mice were infected with adenovirus to express Itch or K393R mutant or GFP (control) in the presence of MG132 as described in panel **a**. Immunoprecipitation was performed using TAp73 antibody, which was immunoblotted for ubiquitin. A significant decrease in ubiquitination of TAp73 was observed in K393R mutant expressing TgAD neurons. Since the same cell lysate was used for co-immunoprecipitation of Itch-myc and TAp73 described in Supplementary Fig. S5A, the input TAp73 Western blot here has also been used for Supplementary Fig. S5A. **e** Rat cortical neurons were treated with A*β*_42_ for 48 h in the presence or absence of JNK inhibitor followed by immunoprecipitation using anti-Itch antibody, Itch-IP was immunoblotted for ubiquitin. A significant decrease in ubiquitination in JNKi-treated cells was observed. **f** Neurons from WT or TgAD mice were infected with adenovirus to express Itch or its S232/T222A mutant or GFP (control) in presence of MG132. Anti-myc antibody was used to IP Itch-myc, which was immunoblotted for ubiquitin. A significant decrease in ubiquitination of the mutant was observed in TgAD neurons. **g** Neurons from WT or TgAD mice were infected with adenovirus to express Itch or S232/T222A mutant or GFP (control) in the presence of MG132. Subsequently, immunoprecipitation was performed using anti-TAp73 antibody, which was immunoblotted for ubiquitin. A significant decrease in ubiquitination of TAp73 with mutant overexpression was observed in TgAD neurons.

Previous studies have indicated that Itch needs to be autoubiquitinated to interact with some of its targets^32,33,41^. However, it is unknown if autoubiquitination is needed for interaction with TAp73 and also sites for autoubiquitination that are responsible for substrate interaction have not been established. Previous proteomics studies have revealed that Itch is ubiquitinated at several sites and most commonly at K393 and K407^42^. Therefore, K393 and K407 mutations were generated and first overexpressed in neuronal PC12 cells. In addition, the catalytic cysteine (C832) which is critical for ubiquitin-ligase activity was also mutated, which resulted in complete abrogation of Itch autoubiquitination (Supplementary Fig. S2A). While there was a dramatic decrease in autoubiquitination of K393R mutant of Itch upon A*β*_42_ treatment, K407R did not reveal a significant change (Supplementary Fig. S2A). These results indicated that K393 may be the major site for autoubiquitination and critical for TAp73 interaction. Therefore, for further studies, adenovirus was prepared to overexpress K393R mutant in neurons. A*β*_42_ caused a dramatic increase in Itch autoubiquitination, which was significantly reduced in K393R mutant (Fig. 4a). Itch autoubiquitination was observed in TgAD neurons and was impaired upon K393R mutation (Fig. 4b). We also assessed if autoubiquitination of Itch influences its interaction with TAp73 by performing co-IP, which revealed a significant reduction in Itch–TAp73 interaction upon K393R mutation in response to A*β*_42_ (Fig. 4c). Similar results were obtained in TgAD neurons as K393R-Itch exhibited reduced interaction with TAp73 (Supplementary Fig. S5). Importantly, TAp73 ubiquitination, which is stimulated in TgAD (Fig. 4d) or A*β*_42_-treated neurons by Itch was significantly reduced by the K393R mutant (Supplementary Fig. S6). These results established that Itch autoubiquitination promotes TAp73 interaction under neurotoxic conditions, which facilitates degradation of the later.

Given that both phosphorylation and autoubiquitination of Itch regulates its interaction with TAp73, we probed if there is a link between these two processes. First, the role of JNK pathway, which regulates Itch phosphorylation, in autoubiquitination was tested. A*β*_42_ induced the autoubiquitination of Itch was significantly reduced in the presence of JNK inhibitor (Fig. 4e). Similarly, treatment of neurons from TgAD animals that exhibited enhanced Itch autoubiquitination was significantly reduced by JNK inhibitor (Supplementary Fig. S4A). Next, the ubiquitination of phospho-deficient mutants was compared to WT Itch. While WT Itch autoubiquitination was significantly enhanced upon A*β*_42_ treatment of cortical neurons (Supplementary Fig. S7A) or TgAD neurons (Fig. 4f), a marked reduction was observed when S232/T222 was mutated to alanine. Concurrently, a significant reversal of TAp73 ubiquitination was also observed in TgAD neurons by this mutant (Fig. 4g), which was also the case in A*β*_42_-treated neurons (Supplementary Fig. S7B). Collectively these studies revealed that the ubiquitination of Itch, which is critical for its interaction with TAp73, is regulated by JNK-mediated phosphorylation of S232/T222. Upon autoubiquitination, Itch interacts with TAp73 resulting in its ubiquitination and degradation.

### Itch phosphorylation and ubiquitination promotes CRNA

**Fig. 5:**
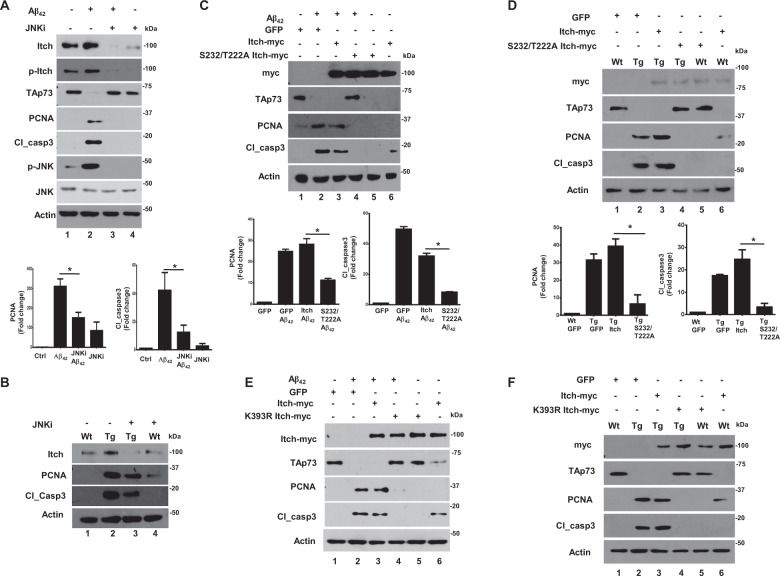
Post-translational modifications of Itch promotes CRNA. **a** Rat cortical neurons were treated with A*β*_42_ for 48 h in the presence or absence of JNKi followed by Western blotting to detect indicated proteins. JNKi prevented PCNA and cl_caspase3 expression, which was quantitated by densitometry (bottom panel, mean ± SEM, *p < 0.05, *t*-test, *N* = 3). **b** Cortical neurons from WT and TgAD animals were treated with JNKi for 48 h followed by Western blotting. A significant decrease in cl_caspase3 and PCNA was observed in JNKi-treated TgAD neurons. **c** Rat cortical neurons were infected with adenovirus to express Itch or its S232/T222A mutant or GFP (control). Subsequently, neurons were treated with A*β*_42_ for 48 h followed by Western blotting for indicated proteins. The levels of PCNA and cl_caspase3 were quantified by densitometry (bottom panel, mean ± SEM, *p < 0.05, ANOVA, *N* = 3). **d** Cortical neurons from WT and TgAD animals were infected with adenovirus to express Itch, S232/T222A mutant, or GFP. Subsequently, Western blotting was performed for indicated proteins. Mutant overexpression prevented cl_caspase3 and PCNA expression in TgAD neurons. The levels of PCNA and cl_caspase3 were quantified by densitometry (bottom panel, mean ± SEM, *p < 0.05, ANOVA, *N* = 3). **e** Rat cortical neurons were infected with adenovirus to express Itch or its K393R mutant or GFP (control). Subsequently, neurons were treated with A*β*_42_ for 48 h followed by Western blotting for indicated proteins. The levels of PCNA and cl_caspase3 were quantified by densitometry (Supplementary Fig. S8A). **f** Cortical neurons from WT and TgAD animals were infected with adenovirus to express Itch or its K393R mutant or only GFP. Subsequently, Western blotting was performed for indicated proteins. K393R-Itch overexpression prevented cl_caspase3 and PCNA expression in TgAD neurons. The levels of PCNA and cl_caspase3 were quantified by densitometry (Suppementary Fig. S8B).

Having established that Itch is regulated by phosphorylation and autoubiquitination by A*β*_42_, it was pertinent to study if these events are critical for CRNA. Since Itch phosphorylation at T222/S232 was mediated by the JNK pathway (Fig. 3b, c), we first tested if this pathway is critical for CRNA. A*β*_42_ treatment resulted in aberrant activation of the JNK pathway, which correlated with high PCNA and cl_caspase3 expression. The treatment with JNKi caused a significant reduction in these proteins, which indicated reversal in CRNA and corroborated well with reduced phosphorylation of Itch (Fig. 5a). Similar experiments were also performed on cortical neurons derived from TgAD animals, which expressed higher PCNA and cl_caspase3 in comparison to WT neurons. The inhibition of JNK also caused a significant reversal of CRNA in TgAD cells (Fig. 5b). These results confirmed that A*β*_42_-induced aberrant activation of JNK pathway causes Itch hyperphosphorylation and CRNA. Given that this phosphorylation occurs at T222 and S232, we next tested direct effects of the phosphorylation on CRNA by using S232/T222A mutant. The overexpression of WT Itch and/or A*β*_42_ treatment causes significant degradation of TAp73 (Fig. 5c, lane 1 vs. lanes 2 and 3) accompanied by CRNA as indicated by increase in cl_caspase3 and PCNA expression. The S232/T222A mutant not only prevented TAp73 degradation but also significantly reverted CRNA (lane 4). Similar observations were obtained in TgAD mouse neurons as significant reversal in CRNA (Fig. 5d, lane 4 vs. lane 3) was caused by the S232/T222A mutant.

Next, the role of Itch autoubiquitination at K393, which is critical for interaction with TAp73 on CRNA was tested. To this end, K393R mutant was used which when overexpressed in cortical neurons prevented TAp73 ubiquitination (Fig. 4d) and degradation (Fig. 5e). Strikingly, this mutant also dramatically reduced PCNA and cl_caspase3 in A*β*_42_-treated cells (Fig. 5e, Supplementary Fig. S8A). These findings were further established in experiments performed on TgAD neurons: K393R mutant almost completely abolished CRNA in these cells (Fig. 5f, lane 4 vs. lane 3 and 2, Supplementary Fig. S8B). Similar results were obtained in neuronal PC12 cells (Supplementary Fig. S2B).

**Fig. 6:**
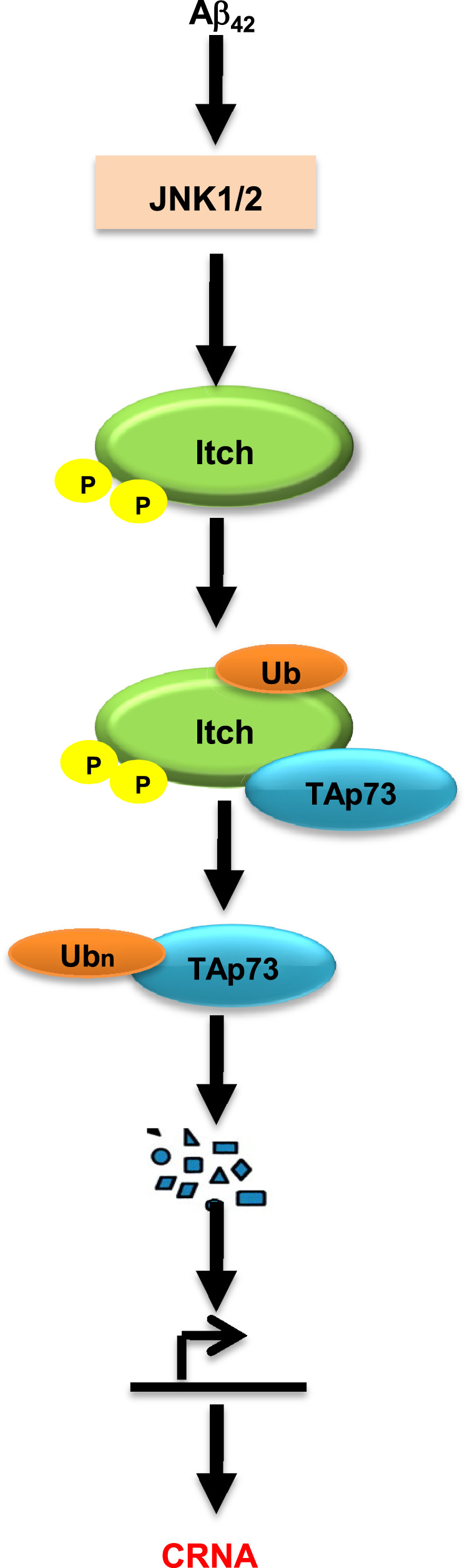
Regulation of Itch in A*β*_42_-induced CRNA. A*β*_42_ stimulates aberrant activation of the JNK pathway, which results in hyperphosphorylation of Itch at T222 and S232 by JNK (Fig. 3). The phosphorylation at these sites promotes its autoubiquitination of Itch at K393 (Fig. 4e and f). These post-translational modifications induce TAp73–Itch interaction (Figs. 3d, e and 4c), which results in TAp73 ubiquitination (Fig. 4d, g) and degradation (Fig. 5c–f) and may affect the transcription of TAp73 target genes. Collectively, these events result in cell cycle re-entry and neuronal apoptosis (CRNA; Figs. 2 and 5).

Present studies delineate a novel pathway triggered by neurotoxic amyloid peptide A*β*_42_, which regulates post-translational regulation of E3 ligase Itch. Aberrant activation of the JNK pathway causes hyperphosphorylation of Itch at S232/T222, which possibly causes a conformational change conducive for its autoubiquitination at K393. As a result, these modifications of Itch promote its interaction with substrates like TAp73 and facilitate its degradation. As a consequence of these events, neurons re-enter the cell cycle and undergo apoptosis, which may contribute to AD pathology (Fig. 6).

## Discussion

Terminally differentiated neurons exit the cell cycle and remain in this state for the remainder of their life, which is critical to maintain their differentiated or arrested state. The reactivation of cell cycle in response to Α*β*_42_ results in their apoptosis. We have demonstrated that the aberrant activation of E3 ubiquitin ligase, Itch deregulates the neuronal cell cycle (Fig. 2), which is expressed in neurons in the steady-state conditions. A*β*_42_ does not cause a significant change in its transcripts (Supplementary Fig. S1) and only a modest change at the protein level (Fig. 1a, b). Its depletion under physiological or steady-state conditions did not cause any neuronal apoptosis and activation of the cell cycle (Fig. 2a, c). These observations seem to suggest that Itch may not have a major physiological function in neurons at least in neuronal survival. In addition, studies on Itchy mice (*Itch*−/−) did not reveal any defects in brain development^27^. There is only one report in which over expression of constitutively active Itch mutants in the brain resulted in enhanced neuronal migration^36^.

Itch is a multi-domain protein with a WW domain, a C2 domain and a PRR in addition to its catalytic HECT domain (Fig. 3a). The WW domain is typically critical for interaction with other proteins^43^ and present studies indicate that the post-translational regulation of Itch in WW domain and the PRR via autoubiquitination and phosphorylation aberrantly activates it in neurons. We established that indeed Itch exhibits significantly enhanced interaction with TAp73 in the presence of A*β*_42_. A previous study also shows that Itch and TAp73 interact which leads to degradation of the later but these studies were performed in cancer and dividing cells and Itch regulation by post-translational modifications was not studied in this report^30^. While a PRR of TAp73 was implicated in their interaction, the underlying mechanism by which Itch regulates TAp73 degradation had remained almost unknown. Itch is regulated by phosphorylation in dividing cells: For instance, ATM phosphorylates it at S161 which causes degradation of c-FLIP-L and c-Jun^44^; phosphorylation at Y371 within the Itch WW domain induced by Src kinase Fyn upon T-cell receptor (TCR) stimulation alters the binding affinity between Itch and JunB and reduces JunB degradation^45^. In addition, JNK1 phosphorylates it at three sites (S199, T222, and S232) within PRR in T-cells^32^, leading to conformational changes that disrupt the self-inhibitory intramolecular interaction between the WW and the HECT domains and increase the catalytic activity of Itch.

Our studies demonstrate that A*β*_42_-induced JNK pathway activation causes aberrant phosphorylation of Itch at S232 and T222, which enhances its interaction with TAp73. While aberrant activation of the JNK pathway contributes to neuronal apoptosis in response to A*β*_42_^46–48^, its regulation of cell cycle machinery has remained largely unknown. We found that JNK pathway contributes to the process of CRNA, as its inhibition reverts this process by preventing phosphorylation of Itch at S232/T222. Interestingly, closely related MEK-ERK pathway did not seem to influence Itch phosphorylation at T222 (Fig. 3b). However, this pathway contributed to the interaction of TAp73 with Itch (Fig. 3d). It is reasonable to suggest that it may directly or indirectly promote phosphorylation of TAp73, which may contribute to its ubiquitination by Itch. MEK-ERK pathway was previously reported to cause TAp73 ubiquitination and degradation^12^. Therefore, it will be interesting to study if TAp73 phosphorylation also contributes to its interaction with Itch in response to A*β*_42_.

We also found that the inhibition of the JNK pathway prevented autoubiquitination of Itch, which is significantly enhanced in response to A*β*_42_ (Fig. 4e). Furthermore, phospho-deficient mutant S232/T222A exhibited dramatic reduction in autoubiquitination (Fig. 4f). The phosphorylation of the PRR region has been suggested to introduce conformational changes that may release Itch from its auto-inhibited state^32^. Our present findings corroborate well with these studies as autoubiquitination was dependent on the phosphorylation of this region. K393 was identified as the primary site as its mutation almost completely abolished autoubiquitination. Importantly, the phospho-deficient mutant T222A/S232A as well as K393R mutant prevented Itch–TAp73 interaction (Figs. 4c, 3f) as well as ubiquitination of TAp73 (Fig. 4d, g).

These results highlighted that the Itch regulation by A*β*_42_ is a tightly regulated process which is dependent on cellular machinery like the activation of a JNK pathway. While TAp73 has also been shown to be a Itch target in dividing cells^30^, these studies were performed in HEK293 or HeLa cells that are very distinct from neurons. Moreover, the mechanism via which Itch is regulated was not dissected in these studies as they were more focussed on p73.

Furthermore, to our knowledge, no neuronal substrates of Itch have been identified. Present findings suggest that Itch may regulate CRNA mainly by targeting TAp73, which was reflected when TAp73 was depleted in Itch-siRNA-transfected cells (Fig. 2d) as reversal of CRNA protection was observed. As reported in the past by us and others^10,12,13^, soluble A*β*_42_ oligomers generated as a result of miscleavage of APP promote aberrant cell cycle re-entry and apoptosis of neurons. It is likely that TAp73 is involved in transcription of genes that suppress the cell cycle progression. Therefore, Itch activation may serve as a key upstream event in this cascade, which prevents this process. Consistent with this, Itch depletion reinstated TAp73 and reversed CRNA (Fig. 2c and d).

Given that CRNA contributes to neuronal loss in AD^7,49,50^, strategies targeting neurons undergoing aberrant cell cycle may be useful. The fact that both K393R and S232/T222A Itch mutants were able to reverse the re-entry of neurons into the cell cycle and apoptosis suggests that these mutants may be used to reverse neurodegeneration or Itch may be targeted in AD neurons for therapeutic purposes.

## Materials and methods

Information related to antibodies and other reagents is provided in Supplementary information.

### Cell culture

Cortical neurons from Embryonic day 18 (E18) Sprague-Dawley rats or Embryonic day 16 (E16) APP/PS1 transgenic AD mice were isolated and cultured as previously published^12,13^. Briefly, E18 rat or E16 mouse embryos were dissected and cortical region of the brain was isolated and treated with Trypsin-DNAse followed by addition of serum-containing media (SCM) and centrifugation at 500 × *g* for 5 min at room temperature. Cell pellet was resuspended in SCM and plated on poly-l-lysine-coated six-well plates. After 12 h, cells were washed with Tyrode’s CMF PBS supplemented with glucose and NaHCO_3_ and were maintained in serum-free medium (SFM) containing B27 and N2 supplement (Gibco, Life technologies), 1× penicillin–streptomycin, L-glutamine, and glucose in 5% CO_2_, for 5 days. Typically, in vitro transfections or A*β*_1-42_ treatments were performed at DIV5.

PC12 (rat pheochromocytoma) cells (ATCC) were maintained in Dulbecco’s modified Eagles medium (DMEM) (Gibco, Life technologies) with 10% heat-inactivated horse serum (Gibco, Life technologies) and 5% heat-inactivated fetal bovine serum (FBS) (Gibco, Life technologies) and Antibiotic/Antimycotic (Gibco, Life technologies). PC12 cells were differentiated in DMEM containing 1% FBS and treated with 50 ng/ml of 2.5 s nerve growth factor (NGF) for 5 days.

HEK293T/A (human embryonic kidney) cells (ATCC) were maintained in DMEM with 10% FBS and 1× antibiotic/antimycotic at 37 °C in 5% CO_2_.

### Transfection and treatment

Lipofectamine 2000 reagent (Invitrogen) was used for transfection of plasmid DNA and siRNA according to manufacturer’s instructions. Cortical neurons and differentiated PC12 cells were transfected with 1–3 μg of plasmid DNA or 100 pmoles of siRNA per well in a six-well plate in SFM without antibiotic. After 3–4 h of transfection, cultures were moved to medium with supplements and antibiotic. Various treatments were typically initiated after 48 h. Adenovirus for GFP (control), Itch or human TAp73*α* (gifted by Dr. Sanjeev Das, NII) was used to overexpress these proteins. 0.5 µM of soluble oligomers of A*β*_1-42_ (R-peptide) was used as described previously^12,13,51^ for 48 h. Typically, cells were treated with 20 μM SP600125/JNKi (Merck) for 48 h, 10 μM U0126 (V112A, Promega) for 48 h and 10 μM MG132 (474790, Merck) for 12 h.

### Immunoblotting

Cells were washed with PBS and lysed using ice cold lysis buffer containing 100 mM Tris–HCl pH 7.4, 5 mM EDTA, 100 mM NaCl, 1% Triton x100, and 10% glycerol, 1 mM phenyl methane sulfonyl fluoride, 1 mM sodium orthovanadate, 20 mM *β*-glycero-phosphate, and 1x protease inhibitor cocktail was added before use. Immunoblotting was performed as described previously^12^ using primary antibodies and secondary antibody conjugated with horse radish peroxidase (HRP). Chemiluminescence reagent West Pico or West Dura (Pierce) was used for detection as per manufacturer’s instructions.

### Immunoprecipitation

Typically, 50–100 μg of protein was incubated with 1 μg of desired antibody for 12 h at 4 °C with shaking in a 250 μl reaction volume. Subsequently, 50 μl of protein A + G Sepharose (Santa Cruz Biotechnology) beads were added to the antibody–protein complex and incubated on a shaker for 5–7 h at 4 °C. The resin was washed at 4 °C to remove unbound proteins and resuspended in lysis buffer and immunoblotting was performed as described above.

### 5-bromo-2′-deoxyuridine (BrdU) incorporation and TUNEL assay

BrdU labeling was performed to detect DNA replication. Anti-BrdU antibody (GE) was used to detect incorporated BrdU^12,13^. Terminal deoxynucleotidyl transferase dUTP nick end labeling (TUNEL) assay to detect cell death was performed by using Dead End fluorometric TUNEL system (G3250, Promega) as per manufacturer’s guidelines and Hoechst 33342 (Molecular Probes) was used to stain the nuclei. These two assays were performed simultaneously and labeled cells were visualized using a Zeiss AxioImager microscope and Axiovision software was used for image acquisition and processing images and population of cells positive for BrdU and/or TUNEL was determined.

### Image and statistical analysis

Image J (NIH) software was for densitometry analysis of desired bands in Western blots. The band intensity of the loading control (Actin) was used for the normalization. Unless indicated otherwise, one-way analysis of variance (ANOVA) or *t*-test was used for statistical analysis (Graph Pad software Inc., USA). Data are represented as mean ± standard error of mean (SEM).

### Animal ethics

All the experiments were designed and performed in accordance with the guidelines of Institutional Ethics Committee. Animal work has been approved by Institutional Animal Ethics Committee with IAEC serial #394/15 and 461/18.

## Supplementary information

Supplementary Information

Supplementary Information

Supp. Fig. S1

Supp. Fig. S2

Supp. Fig. S3

Supp. Fig. S4

Supp. Fig. S5

Supp. Fig. S6

Supp. Fig. S7

Supp. Fig. S8

Supplementary Table S1
