# Supplementary material for: Aberrant activation of neuronal cell cycle caused by dysregulation of ubiquitin ligase Itch results in neurodegeneration
Source: Cell Death Dis. 2020 Jun 8;11(6):441. doi: 10.1038/s41419-020-2647-1 (PMC7280246; doi:10.1038/s41419-020-2647-1)
Supplement: Supplementary file 1 — Supplementary Information [file 41419_2020_2647_MOESM1_ESM.docx]

**Supplementary Information**

**Supplementary Methods**

**Materials:**

**Antibodies:** Antibodies were used against the following proteins: cyclin D1 (sc-753, 1:500), PCNA (sc-56, 1:500), actin (sc-47778, 1:2000), Ubiquitin (sc-7383, 1:1000), HA probe F-7 (sc-7392, 1:500), phospho-ERK (sc-7383, 1:500), ERK (sc-94, 1:1000), JNK-1(F3) (sc-1648, 1:500), phospho-JNK (G-7) (sc-62548, 1:500) were from Santa Cruz Biotechnology, Santa Cruz, CA. Antibody against cleaved caspase 3 (D-175) (#9661, 1:500), Itch (D8Q6D) (#12117, 1:1000) and myc-tag (9B11) (#2276, 1:2000) were from Cell Signaling technology. Antibodies against TAp73 (5B429) (NBP2-24737, 1:2000) from Novus, phospho-T222 Itch (AB10050, 1:2000) from EMD Millipore Corp., anti-GFP (11814460001, 1:2000) from Roche Diagnostics and anti-BrdU (RPN202, 1:50 for IFA) from GE Healthcare Bio-Sci­ences, Piscataway, NJ.

**siRNA:** *Itch* siRNA duplex of the following sequence was custom synthesized by Invitrogen for efficient knock down:

Itch siRNA: 5’- GAGAAGAAGGUUUAGAUUAUU -3’;

scrambled siRNA (scr siRNA): 5’- GAGGAAAAGUGUGAUUUAAUU-3’.

**shRNA:** For Itch knockdown using shRNA in rat primary cortical neurons, Itch shRNA was overexpressed using pSuperneo plasmid. Itch shRNA (short hairpin RNA) and a scrambled shRNA were designed as a stem loop structure using oligonucleotides with *EcoRI* and *PacI* overhangs. The annealed oligos were ligated between *BglII* and *HindIII* site in pSuperneo vector:

Itch shRNA Forward: 5'GATCCCCGAGAAGAAGGTTTAGATTATTCAAGAG ATAATCTAAACCTTCTTCTCTTTTTA 3'

Itch shRNA Reverse: 5'AGCTTAAAAAGAGAAGAAGGTTTAGATTATCTCTT GAATAATCTAAACCTTCTTCTCGGG3'

Scrambled Itch shRNA Forward: 5' GATCCCCGAGGAAAAGTGTGATTTAATTCAA GAGATTAAATCACACTTTTCCTCTTTTTA 3'

Scrambled Itch shRNA Reverse: 5' AGCTTAAAAAGAGGAAAAGTGTGATTTAATCT CTTGAATTAAATCACACTTTTCCTCGGG 3'

For knockdown of p73 in rat primary cortical neurons, a rat TAp73 specific shRNA was over expressed using a lentivirus. TAp73 shRNA and a scrambled shRNA were designed from DNA binding domain as a stem loop structure using following oligonucleotides with *EcoRI* and *PacI* overhangs. The annealed oligos were ligated between *EcoR1* and *PacI* site in pLKO.3G vector:

p73 shRNA Forward: 5’ AATTCAGACATGCCCCATCCAGATCTCGAG ATCTGGATGGGGCATGTCTTTTTTTTAAT 3’

p73 shRNA Reverse: 5’ TAAAAAAAAGACATGCCCCATCCAGATCTCGAG ATCTGGATGGGGCATGTCTG 3’

Scrambled p73 Forward: 5’ AATTCAGACCGTACTCCCCAAGATCTCGAG ATCTTGGGGAGTACGGTCTTTTTTTTAAT 3’

Scrambled p73 Reverse: 5’ TAAAAAAAAGACCGTACTCCCCAAGATCTCGAG ATCTTGGGGAGTACGGTCTG 3’.

**Plasmid DNA constructs**:

TAp73α-HA pcDNA 3.0, Itch-myc pCIneo constructs (Addgene #2859) were used for overexpression mainly in neuronal PC-12 cells. For generation of Itch mutants, Itch-myc pCIneo plasmid was used as template and mutations were incorporated by site directed mutagenesis using Phusion DNA polymerase (FNZ530, Thermo) and PCR primers indicated in Table S1.

For overexpression of Itch and its mutants in primary cortical neurons, WT Itch or its mutants with a C-terminal myc tag were subcloned from in pAdTrack-CMV adenoviral shuttle vector within *KpnI* and *NotI* restriction sites. GFP was present in this vector and independent promoter was used to drive its expression.

**Adenovirus generation and transduction**

Itch-myc-pAdTrack shuttle vector construct was digested with *PmeI,* and elec­troporated in *Escherichia coli* BJ5138 containing pAdEasy-1 vector. The recombinant clones were digested with *Pac* I and transfected in HEK293A cells. The adenovirus was harvested and amplified using standard proce­dures. Virus at ~ 10 multiplicity of infection (MOI) was incubated with cortical neurons and efficiency of infection was determined by observing GFP fluorescence after 24-48 h.

**Alzheimer’s disease Mouse Model**

Amyloid Precursor Protein (APP)/ Presenillin 1 (PS1) transgenic (Tg) mouse model for AD (strain name B6C3-Tg APPswe, PSEN1dE9 85Dbo/J; stock number 004462) maintained by the Jackson laboratory was gifted by National Brain Research Centre, Manesar to NII, New Delhi. These mice are double transgenic for amyloid-β precursor protein (APP)/ presenilin 1 (PS1) as they express a chimeric mouse/human amyloid-β precursor protein containing the K595N/M596L Swedish mutations and a mutant human presenilin 1 carrying the exon 9-deleted variant under the control of mouse prion promoter elements, directing transgene expression predominantly to the central nervous system neurons. The levels of Aβ_42_ produced are significantly higher in these animals (Jankowsky et al, 2004; Jankowsky et al, 2001). Wild type and transgenic (TgAD) mice were genotyped using genomic DNA isolated from mouse tail.

**Quantitative real-time PCR**

Total RNA was extracted from cells using TRIzol reagent (10291060, Life technologies) or RNeasy mini kit (74106, Qiagen). The cDNA was synthesized using random hexamers (SO142, Thermo Scientific) and RevertAid H minus reverse transcriptase (EP0451, Thermo Scientific) using 1μg of total RNA. Quantitative RT-PCR reactions were performed in triplicate for each sample using Real Time Cycler (Eppendorf) and Dynamo Color Flash SYBR Green PCR Master Mix (F-416L, Thermo Scientific). Reactions were carried out in 20 μL following manufacturer’s instructions. Primers with following sequence were used:

5’ TGTAGAAGTCACAGTAGATGGGC3’ and

AGGGGTAACGATAACTGTGAGG for Itch;

5'ATGGGAGTTGCTGTTGAAGTCA and

5’ CCGAGGGCCCACTAAAGG 3’ for GAPDH (control). Quantification was done using the 2^(−ΔΔCT)^ method.
