# Supplementary material for: Aberrant activation of neuronal cell cycle caused by dysregulation of ubiquitin ligase Itch results in neurodegeneration
Source: Cell Death Dis. 2020 Jun 8;11(6):441. doi: 10.1038/s41419-020-2647-1 (PMC7280246; doi:10.1038/s41419-020-2647-1)
Supplement: Supplementary file 2 — Supplementary Information [file 41419_2020_2647_MOESM2_ESM.doc]

**Supplementary Figure legends**

**Fig. S1:** Rat cortical neurons were left untreated or treated with Aβ_42_ for 48h (A) or cortical neurons were cultured from wild type (WT) or APP/PS1 Tg (TgAD) mice (B). Subsequently, RNA was isolated and cDNA was prepared using reverse transcriptase which was used for Real-Time PCR to quantitate the expression of Itch was normalized with respect to GAPDH (mean±SEM, ANOVA, N=3, not significant (ns)).

**Fig. S2** A. Neuronal PC12 cells were transfected with Wild Type, K393R, K407R or C832A mutants of Itch, which were myc-tagged, in presence/ absence of Aβ_42_ followed by MG132 treatment. After 48h, anti-myc antibody was used to IP Itch or its mutants followed by Western blotting with anti-TAp73 or anti-ubiquitin antibody. Total protein lysates (input) were also used for Western blotting with indicated antibodies.

B. Neuronal PC12 cells were transfected with indicated plasmids for Itch and its mutants followed by Aβ_42_ treatment as described in panel A followed by Western blotting using indicated antibodies.

**Fig. S3** Cortical neurons from WT and TgAD animals were transfected with Itch or its S232/T272A mutant. Western blotting was performed with anti-phospho Itch and other indicated antibodies.

**Fig. S4**. A. Cortical neurons from WT and TgAD animals were treated with JNKi for 48h in the presence of MG132. Itch was immunoprecipitated and IP was subjected to Western blotting with anti-ubiquitin antibody. Total cell lysate (input) was also probed for Itch.

B. Rat cortical neurons were treated with Aβ_42_ for 48h in the presence or absence of JNK inhibitor followed by immunoprecipitation using anti-Itch antibody, and IP was immunoblotted with anti-JNK1 antibody.

**Fig. S5.** A. Neurons from WT or TgAD mice were infected with adenovirus to express Itch or K393R mutant or GFP (control) in the presence of MG132 as described in figure 4D. Immunoprecipitation was performed using anti-myc-antibody, which was immunoblotted for TAp73. The very same lysate was used for IP of TAp73 followed by anti-ubiquitin Western blot, which is provided in Figure 4D. Since the same lysate was used for both immunoprecipitations, the same TAp73 Western blot for input has also been used in Figure 4D.

B. Densitometry of bands corresponding to TAp73 in Itch-IP (panel A) was performed for quantitation of interaction and fold change was determined with respect to wild type Itch transfected cells (Mean ±SEM, * p<0.05, t-test, N=3).

**Fig. S6**. Rat cortical neurons were treated with Aβ_42_ and infected with adenovirus to express Itch or K393R mutant or GFP (control) in the presence of MG132. Immunoprecipitation was performed using TAp73 antibody, which was immunoblotted for ubiquitin.

**Fig. S7 A and B**. Rat cortical neurons were treated with Aβ_42_ and infected with adenovirus to express Itch or its S232/T222A mutant or GFP (control) in the presence of MG132. Anti-myc antibody (A) was used to IP Itch-myc or anti-TAp73 antibody to IP TAp73 (B) and the IPs were immunoblotted for ubiquitin.

**Fig. S8.** Densitometry analysis for Western blots presented in Fig. 5E (A) and Fig. 5F (B). The levels of PCNA and cl_caspase3 were quantified by densitometry (Mean ± SEM, * p<0.05, ANOVA, N=3). K393R mutant suppressed PCNA and cl_caspase3 expression in both cases.
