# Supplementary figures and images for: Aberrant activation of neuronal cell cycle caused by dysregulation of ubiquitin ligase Itch results in neurodegeneration
Source: Cell Death Dis. 2020 Jun 8;11(6):441. doi: 10.1038/s41419-020-2647-1 (PMC7280246; doi:10.1038/s41419-020-2647-1)

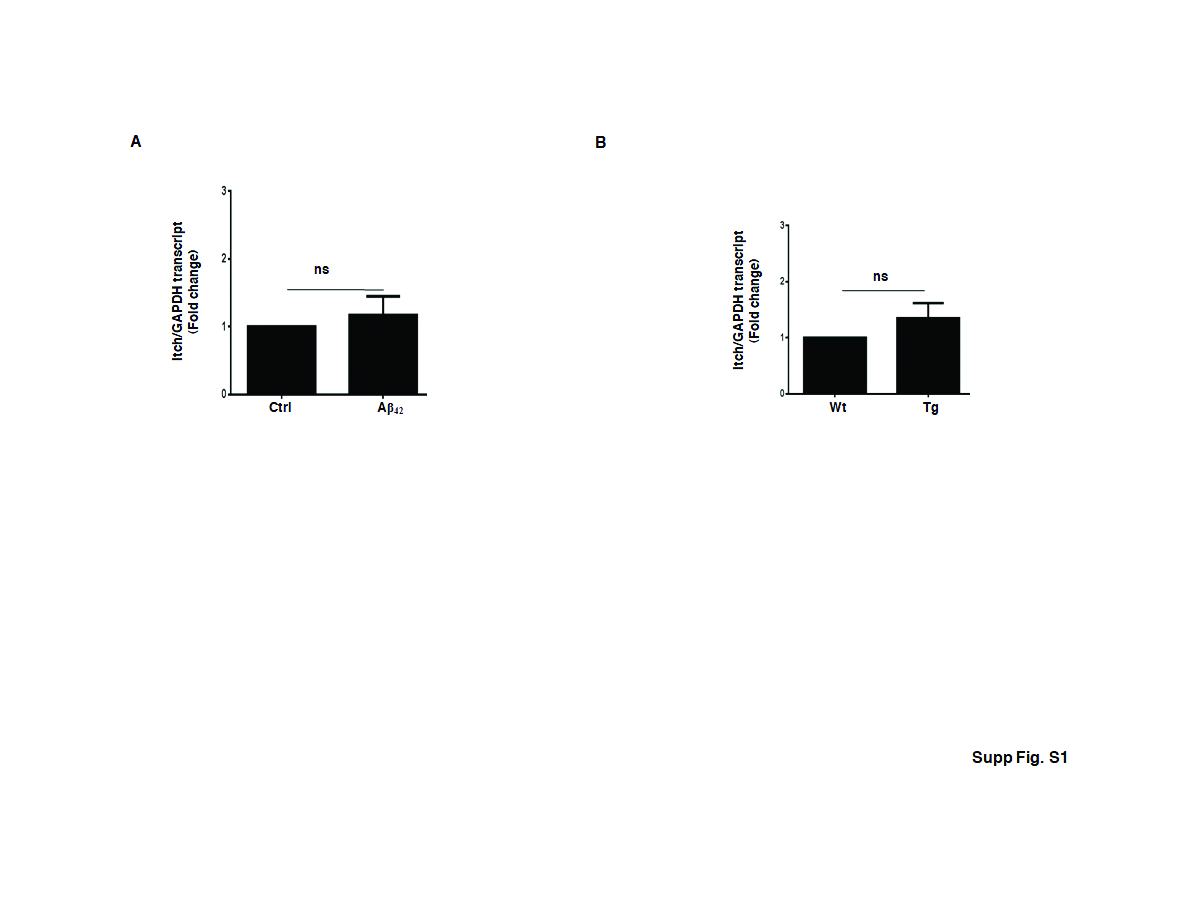

Supplement: Supplementary file 3 — Supp. Fig. S1 [file 41419_2020_2647_MOESM3_ESM.tif]

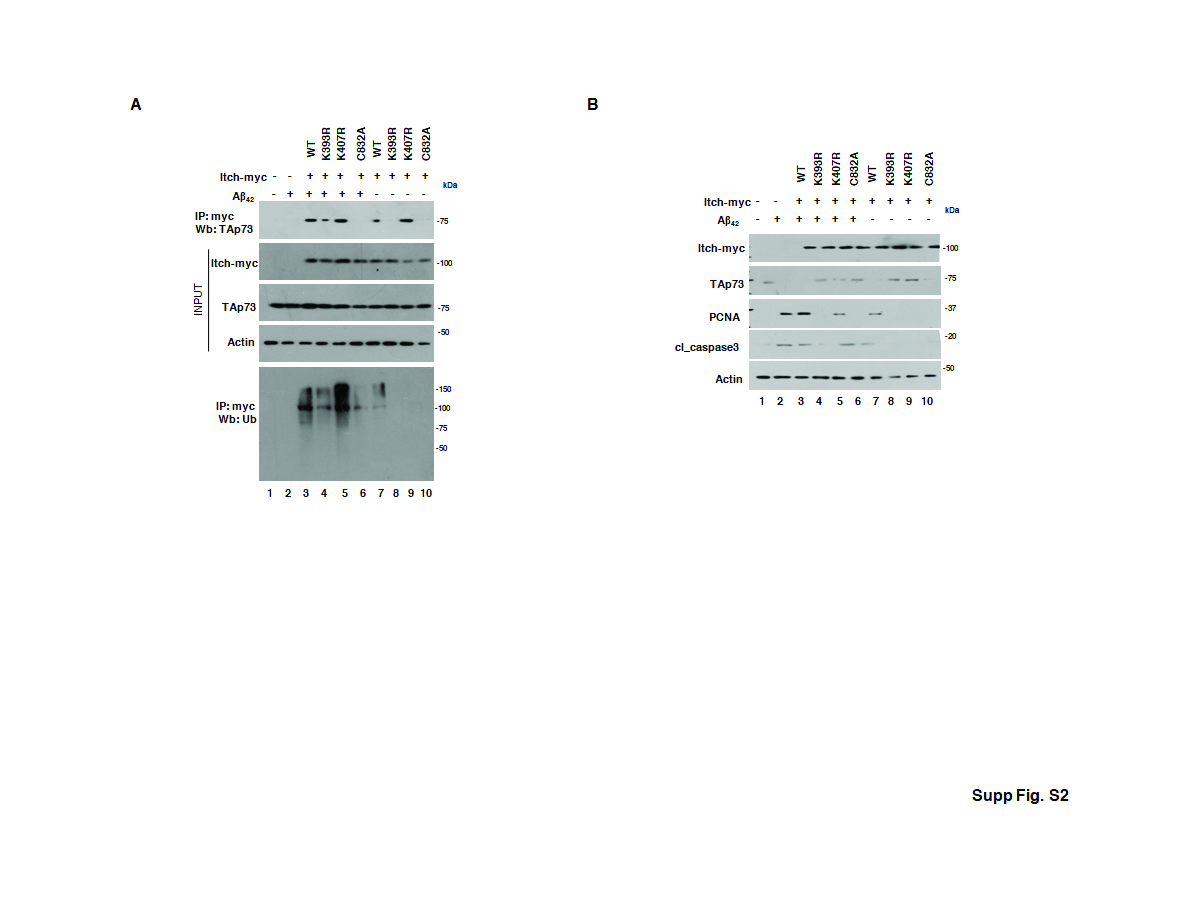

Supplement: Supplementary file 4 — Supp. Fig. S2 [file 41419_2020_2647_MOESM4_ESM.tif]

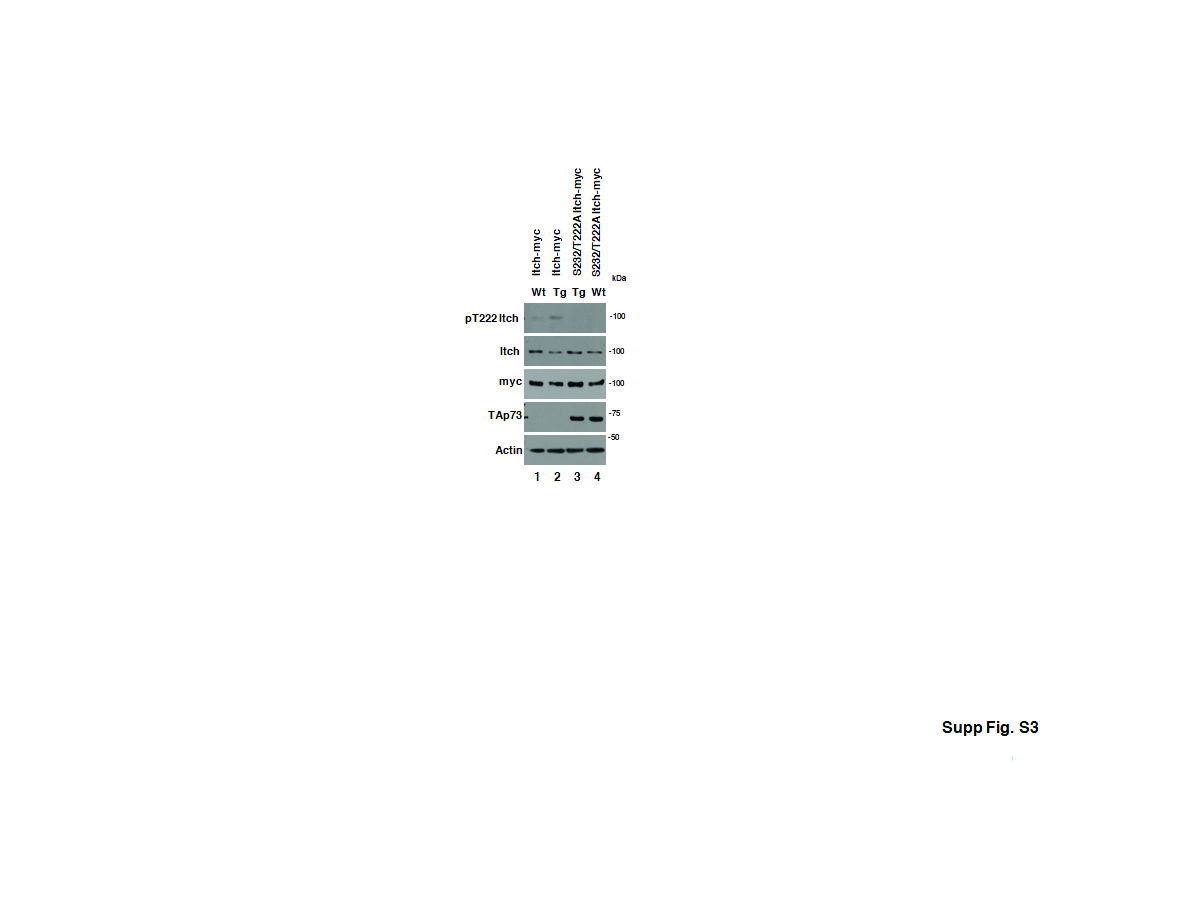

Supplement: Supplementary file 5 — Supp. Fig. S3 [file 41419_2020_2647_MOESM5_ESM.tif]

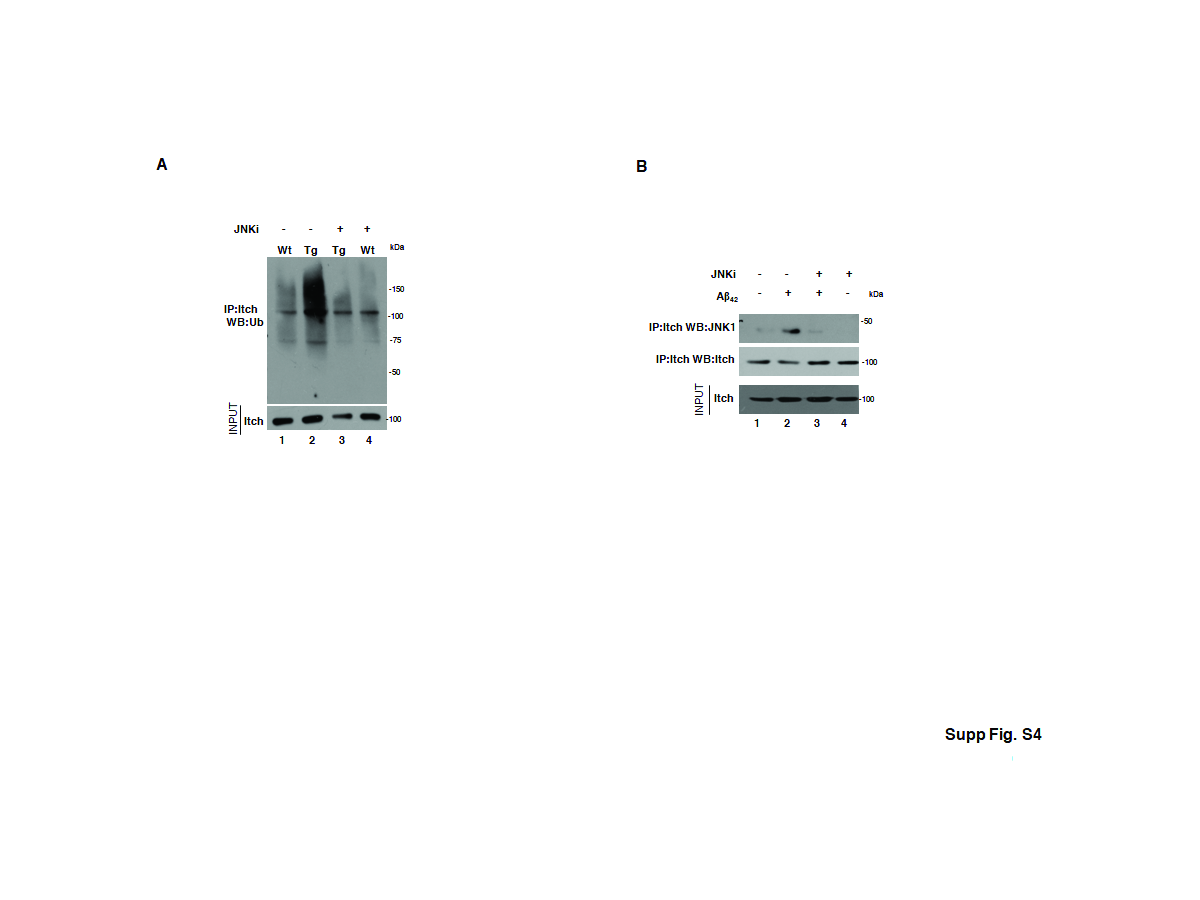

Supplement: Supplementary file 6 — Supp. Fig. S4 [file 41419_2020_2647_MOESM6_ESM.tif]

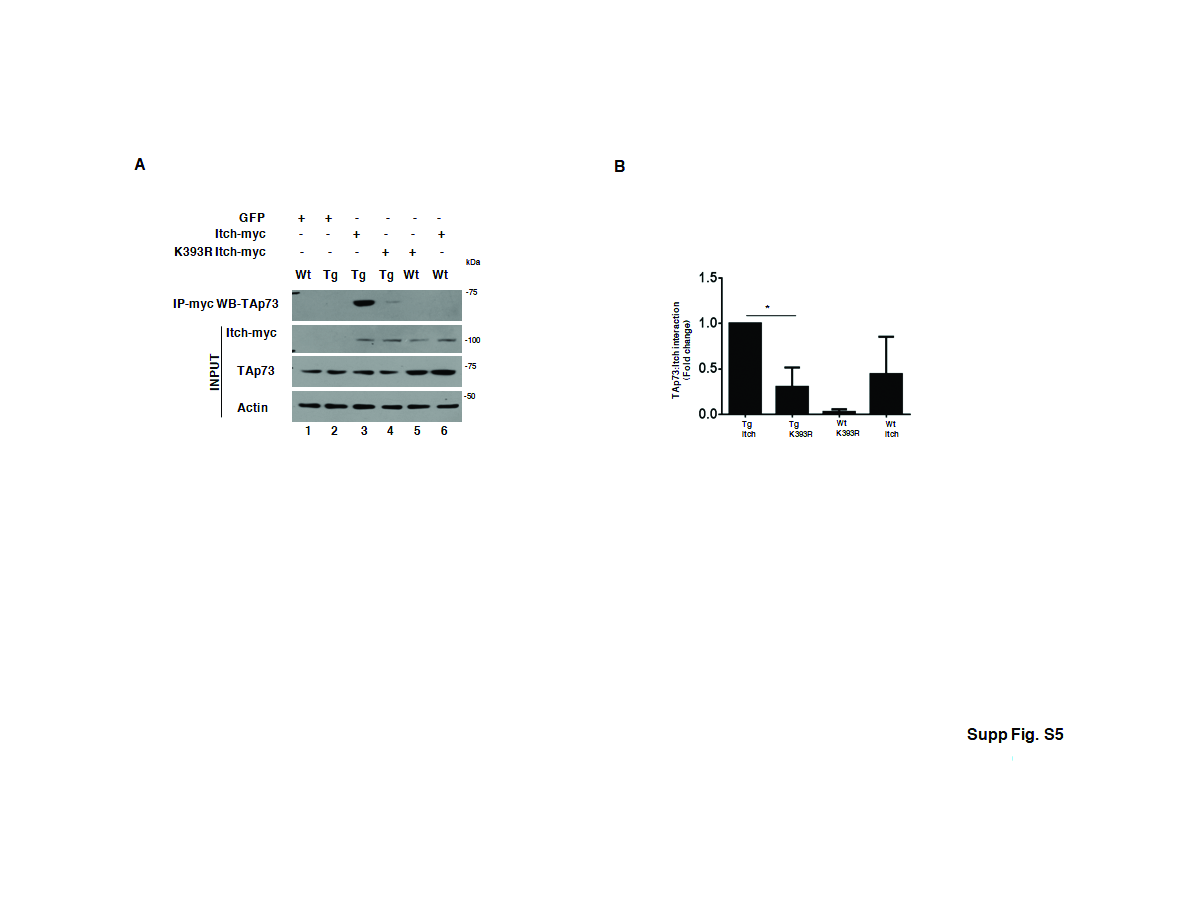

Supplement: Supplementary file 7 — Supp. Fig. S5 [file 41419_2020_2647_MOESM7_ESM.tif]

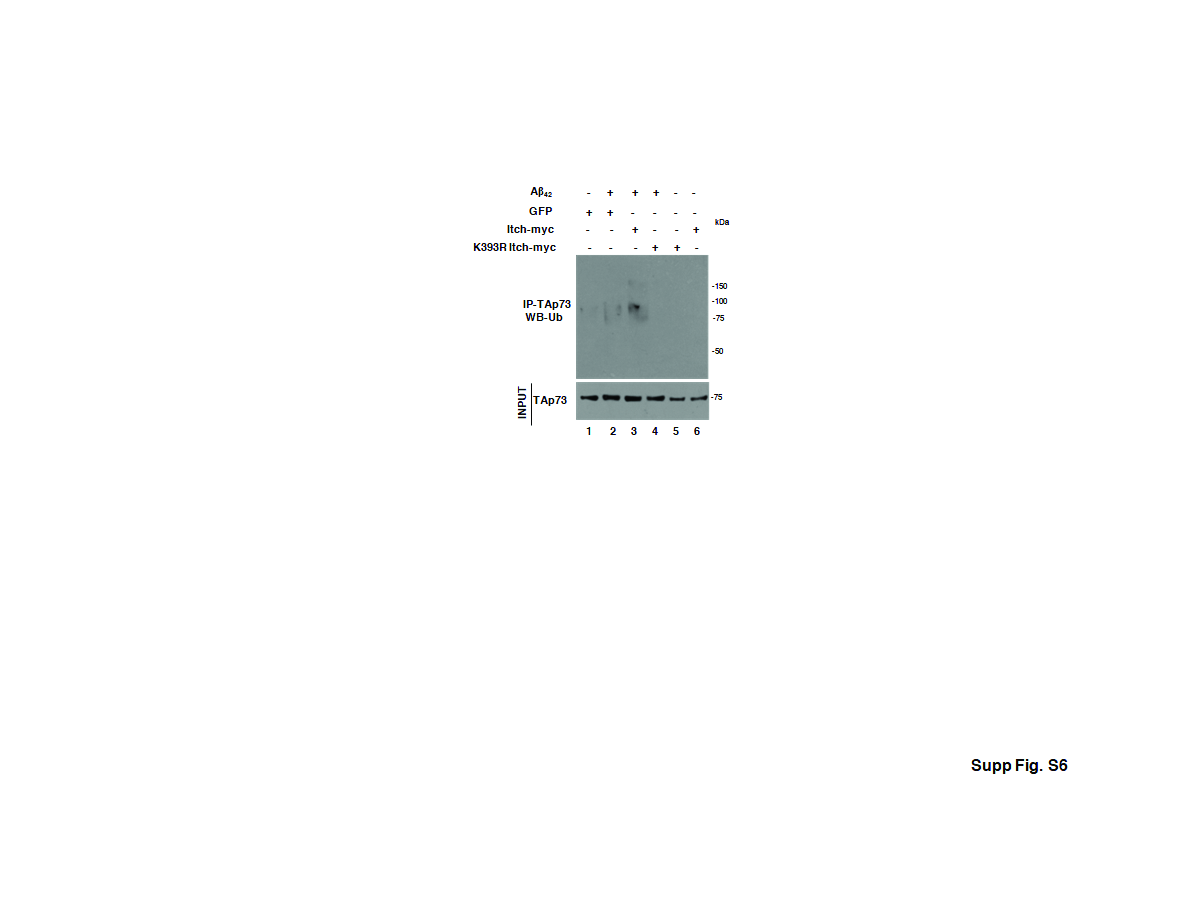

Supplement: Supplementary file 8 — Supp. Fig. S6 [file 41419_2020_2647_MOESM8_ESM.tif]

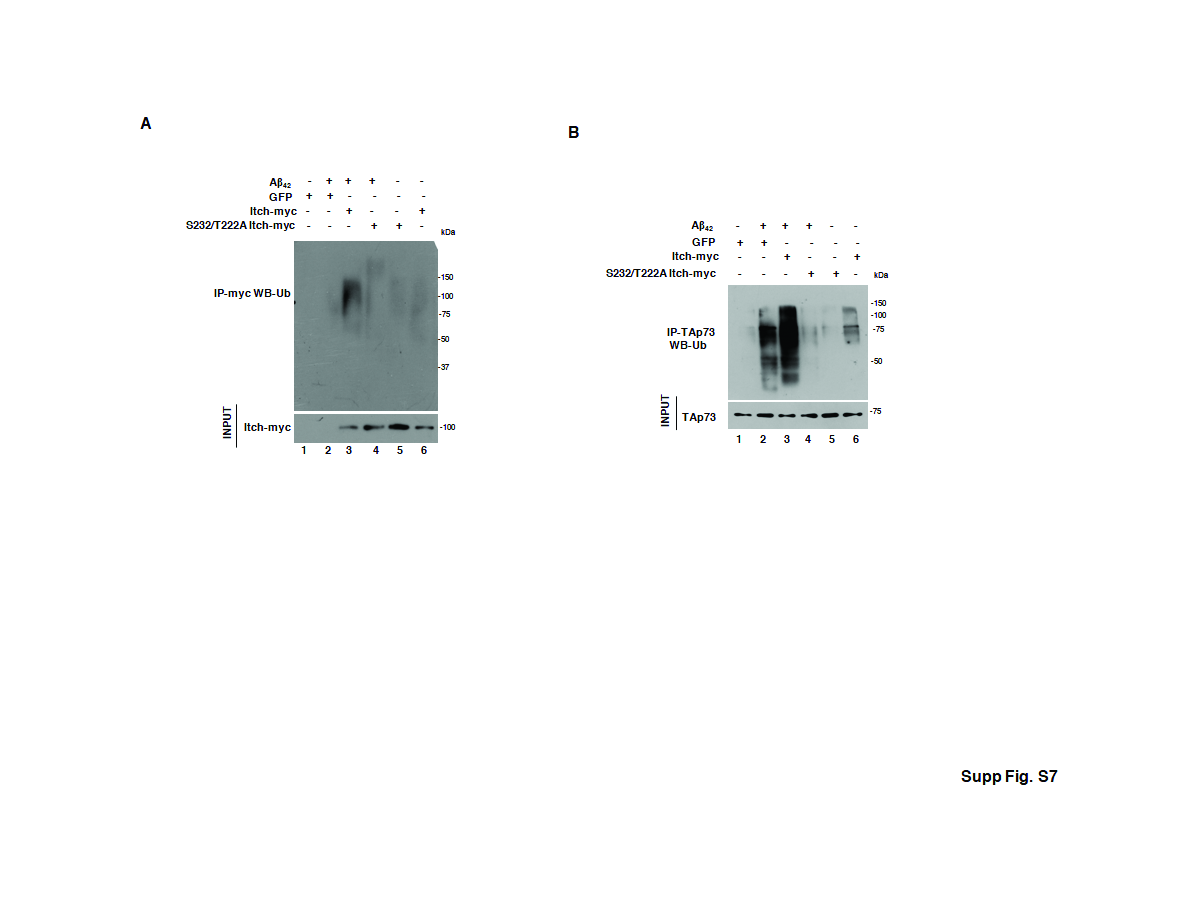

Supplement: Supplementary file 9 — Supp. Fig. S7 [file 41419_2020_2647_MOESM9_ESM.tif]

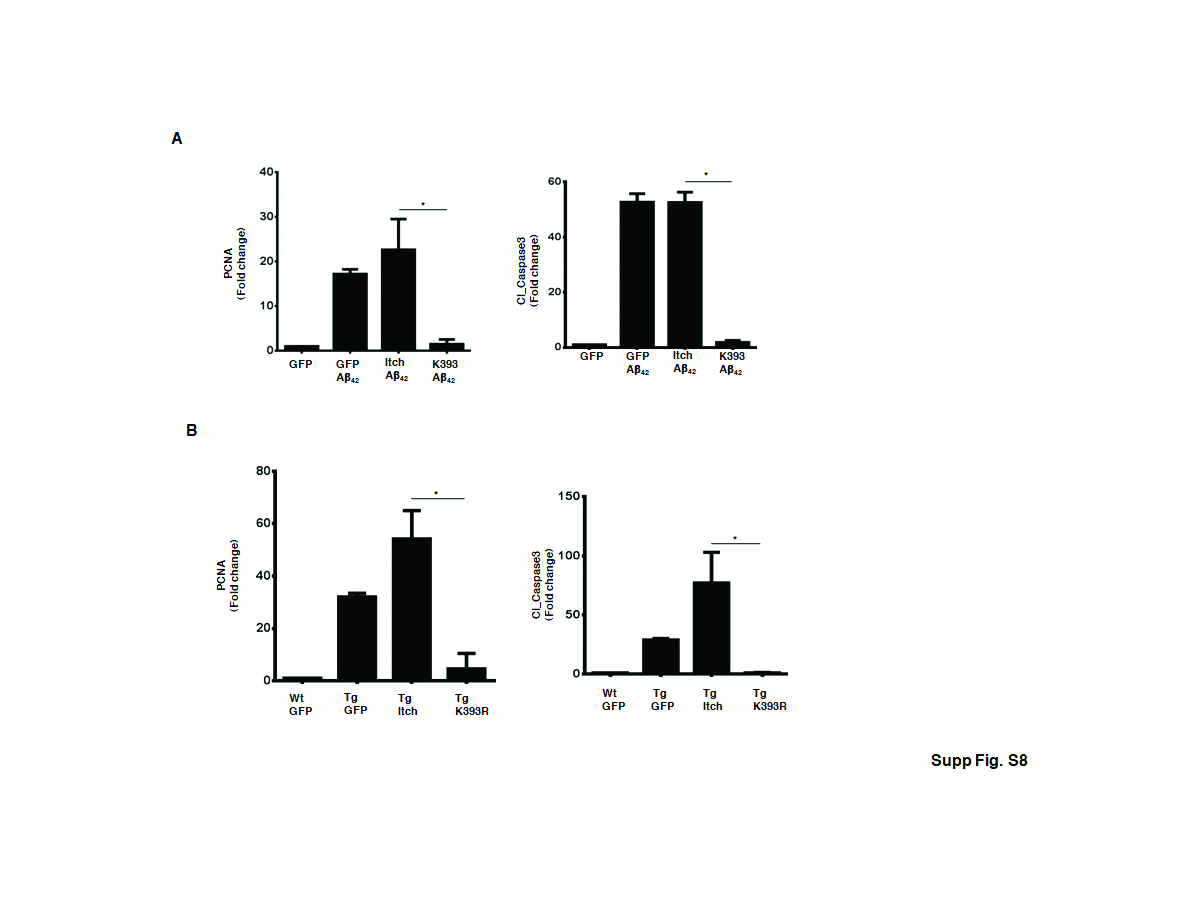

Supplement: Supplementary file 10 — Supp. Fig. S8 [file 41419_2020_2647_MOESM10_ESM.tif]
