# Supplementary material for: Aberrant activation of neuronal cell cycle caused by dysregulation of ubiquitin ligase Itch results in neurodegeneration
Source: Cell Death Dis. 2020 Jun 8;11(6):441. doi: 10.1038/s41419-020-2647-1 (PMC7280246; doi:10.1038/s41419-020-2647-1)
Supplement: Supplementary file 11 — Supplementary Table S1 [file 41419_2020_2647_MOESM11_ESM.doc]

**Table S1. PCR primers used for various plasmid DNA constructs**

| **Oligo** | **Sequence** | **Modification/mutation** |
| --- | --- | --- |
| T222A | Forward_CCACCTCCACCTGCTCCAAGAAGACCA  Reverse_ TGGTCTTCTTGGAGCAGGTGGAGGTGG | Itch PRR T222-A (ACC-GCC) |
| S232A | Forward_ GCTTCTGTCAATGGCGCACCATCCACGAATTCT  Reverse_ AGAATTCGTGGATGGTGCGCCATTGACAGAAGC | Itch S232-A (TCA-GCA) |
| S199A | Forward_AGGGCCAATGGGAACAATGCTCCGTCTCTTTCAAATGGT  Reverse_ ACCATTTGAAAGAGACGGAGCATTGTTCCCATTGGCCCT | Itch S199-A (TCT-GCT) |
| C832A | Forward_AGAAGCCATACTGCGTTTAACCGCCTGGAC  Reverse_GTCCAGGCGGTTAAACGCAGTATGGCTTCT | Itch C832-A (ACA- CGC) |
| K393R | Forward_ GCTACCTCACAGAACAGAGAATTTGATCCGCTT  Reverse_ AAGCGGATCAAATTCTCTGTTCTGTGAGGTAGC | Itch K393-R (AAA- AGA) |
| K407R | Forward_ CCCCCTGGATGGGAGAGGAGAACTGATAGCAAC  Reverse_ GTTGCTATCAGTTCTCCTCTCCCATCCAGGGGG | Itch K407R to A (AAG-AGG) |
